# Bacterial Diversity Dynamics in Sandy Loam Soils in Tanzania Under Varying Fertilizer-Derived Uranium Concentrations

**DOI:** 10.3390/microorganisms13081886

**Published:** 2025-08-13

**Authors:** Dennis A. Mwalongo, Jacob B. Lisuma, Nils H. Haneklaus, Ali Maged, Hendrik Brink, Fernando P. Carvalho, Stanisław Wacławek, Nelson Mpumi, Aloyce I. Amasi, Jerome M. Mwimanzi, Furaha M. Chuma, Thomas T. Kivevele, Kelvin M. Mtei

**Affiliations:** 1 Tanzania Atomic Energy Commission (TAEC), Arusha P.O. Box 743, Tanzania; 2 Tobacco Research Institute of Tanzania (TORITA), Tabora P.O. Box 431, Tanzania; 3 Nelson Mandela African Institution of Science and Technology (NM-AIST), School for Materials, Energy, Water, Environmental Science and Engineering, Arusha P.O. Box 447, Tanzania; 4 Td-Lab Sustainable Mineral Resources, University for Continuing Education Krems, 3500 Krems, Austria; 5 Unit for Energy and Technology Systems—Nuclear Engineering, North-West University, 11 Hoffman Street, Potchefstroom 2520, South Africa; 6 Geology Department, Faculty of Science, Suez University, Suez 43518, Egypt; 7 Department of Chemical Engineering, University of Pretoria, Pretoria 0028, South Africa; 8 Laboratório de Protecção e Segurança Radiológica, Instituto Superior Técnico/Campus Tecnológico Nuclear, Universidade de Lisboa, Lisboa, Portugal; 9 Institute for Nanomaterials, Advanced Technologies, and Innovation, Technical University of Liberec, Liberec, Czech Republic

**Keywords:** uranium, soil bacterial diversity, loam soil, phosphate fertilizers, Tanzania

## Abstract

The presence of radiotoxic uranium (U) in mineral fertilizers is of global concern. A pilot study was conducted in Tabora (Tanzania) to determine the release of U from three brands of phosphate fertilizers and its impact on soil bacteria. The experiment used three types of fertilizer: Minjingu Powder (MP), Nafaka Plus (NP), a mixed and granulated fertilizer made from Minjingu Phosphate Rock (MPR), and YaraMila Cereal (YC) fertilizer. There was also a control treatment that was not fertilized (NF). Alpha diversity and the R tool were used to analyze bacterial diversity in four samples within an average sequencing depth of 74,466 reads, using metrics like ASVs, Shannon index, and Chao1. The results showed that the number of amplicon sequence variants (ASVs) in the DNA from soil bacteria decreased, specifically to 400 ASVs, in the NP treatment, which was in line with the higher U concentration (3.93 mg kg^−1^) in the soils. In contrast, the MP fertilizer treatment, associated with a lower U concentration (3.06 mg kg^−1^) in soils, exhibited an increase in ASVs within the DNA of soil bacteria, reaching 795; the highest ASV value (822) was observed in the NF treatment. Higher amounts of U in the soil plots seemed to have resulted in more types of bacteria, with the *Actinobacteriota* phylum being the most common in all of the treatments. The NP (3.93 mg kg^−3^ U concentration) and MP (3.06 mg kg^−3^ U concentration) treatments were the only ones that showed *Halobacteriota* and *Crenarchaeota* phyla. Nonetheless, bacterial diversity may also account for the alterations in soil phosphorus and nitrogen following fertilizer application. The YaraMila Cereal treatment did not seem to be linked to any particular bacterial phylum. This means that in this study it did not have any measurable effect on the soil bacteria species compared to the MP and NP treatments.

## 1. Introduction

Maize (*Zea mays* L.) is an essential food crop in Tanzania that contributes significantly to National Food Security. Small-scale farmers across the country cultivate more than 95% of the maize [1]. In Tanzania, maize production requires the use of fertilizers to obtain satisfactory crop yields. The fertilizers commonly used are rich in nitrogen (N), phosphorus (P), and potassium (K), with smaller amounts of micronutrients like boron (B), copper (Cu), and zinc (Zn) [2]. Farmers in Tanzania have been using these NPK (nitrogen, phosphorus, and potassium) fertilizers for over a decade now.

Recently, new fertilizers aimed to suit better maize crops, such as YaraMila Cereal (YC) and Nafaka Plus (NP), have been developed and introduced for use in Tanzania. The YaraMila Cereal (YC) fertilizer was developed by the Yara Fertilizer Company and is imported into Tanzania, while NP is produced locally by the Minjingu Mines and Fertilizer Limited company, located in northern Tanzania. Tanzania imports the YC fertilizer containing P, but it may also contain trace amounts of uranium (U) [3]. The Nafaka Plus (NP) fertilizer is a product from Tanzania Minjingu phosphate ore. Minjingu phosphate ore shows elevated concentrations of naturally occurring U if compared to other phosphate ores [4,5,6]. As an environmental pollutant, naturally occurring U can cause a number of ailments, including cancer, dementia, leukemia, renal failure, and neurasthenia, among others, when it enters the human body [7].

Most Tanzanian farmers are using the YC fertilizer now, while the majority of maize farmers, especially in the north of the country, are using NP fertilizers for maize production. However, based on the high quality of Minjingu phosphate ore, some farmers have also still been using Minjingu Powder (MP), natural phosphate rock milled and ground into fine powder form that is then directly applied on soil to enhance maize crop production. Researchers have reported that high U levels in soils can alter the structure and activity of bacterial communities, leading to changes in soil bacterial diversity [8,9,10,11,12,13,14]. Some studies found that soils with increased U contamination had the highest levels of bacterial diversity, with the most common bacteria genera being *Firmicutes*, *Proteobacteria*, *Acidobacteria*, and *Actinobacteria* [15,16,17,18]. Additionally, a recent study found that the *Bacteroidota* (*Bacteroidetes*) group is also common in soils contaminated with U [19].

Repeated application of phosphate fertilizers with elevated U content can enhance the concentration of this radiotoxic element in soils over longer periods of time [20,21,22]. Increased levels of U in soils were also found to be associated with a decreased ability of microorganisms to utilize carbon sources [23]. An investigation near a U mine in Australia reported, for instance, that the *Proteobacteria* phylum was more abundant in soils with higher U concentrations, ranging from 2 to 900 mg U kg^−1^. Although about 48% of bacteria have not been identified, the new bacteria developed in these soils with higher U concentrations are of increased interest [24]. These findings suggest that the presence of bacteria from the *Proteobacteria* group, which is linked to higher U levels, could indeed be a useful tool to indicate if relevant U contamination is present in a specific soil [25]. Minjingu phosphate fertilizers can have relatively high U levels, in the range of 300–400 mg kg^−1^ [6]. The influence of U on bacterial activity has thus far only been investigated for soils near mines (usually uranium mines), mine wastes, or U processing sites. The presence of naturally occurring U in mineral fertilizers is gaining increased attention due to potential environmental risks [26], the potential use of this U as a recoverable unconventional resource [27,28,29,30], and nuclear non-proliferation concerns [31,32]. The relatively high concentrations of naturally occurring U in Minjingu phosphate ore are known and are recognized. To the best of our knowledge, no research has investigated the impact of varying U concentrations in fertilizers on bacterial diversity in Tanzania or elsewhere yet. This study elucidates the effects of commercial phosphate fertilizers with elevated U content on soil bacterial communities and identifies cereal fertilizers that exert minimal influence on them.

## 2. Materials and Methods

### 2.1. Location of the Study Area and Collection of Soil Samples

The experiments were conducted in Tumbi, Tabora Region, Mid-Western Tanzania (GPS coordinates: 5°30′44.4″ S, 32°40′7.40″ E; 1151 m a.s.l.), on sandy loam soil with very low organic carbon (0.16%) and low calcium (0.10 Cmol(+)kg^−1^) during the 2021–2022 cropping season (typically, planting takes place from October to December and harvest from March to May). The land had remained uncultivated for over a decade before the study. The recorded mean air temperature and total rainfall during the cropping season were 27 °C and 952 mm, respectively.

Before the experiments, we collected soil samples from each plot using a soil core to determine the inherent U concentrations and soil pH. Following the experiments, we extracted soil samples from each plot’s maize rhizosphere using soil cores. We considered soils detached from roots as rhizospheres and used them to determine U concentrations and soil pH. Each treatment was applied to a 6 m × 6 m plot, with a spacing of 0.75 m between ridges and 0.30 m between plants. The maize variety used was DKC8053, sown on 15 November 2021, and collected on 10 April 2022. The experiment consisted of four maize planting treatments (M), each replicated three times. Table 1 presents an overview of the treatments and fertilizer compositions [33] used in the study.

The NF + M treatment plot, designated as a control plot, did not receive any fertilizer treatment and is sufficiently far away from the other plots to assure that this also did not happen unintentionally. Maize plants in the other three treatments received 5 g fertilizer per plant in three equal applications 2, 4, and 8 weeks after sowing. Sixteen soil samples were collected between the plants using an Auger sampler inserted in the soil to a depth of 20 cm below the surface. Rhizosphere soil (soil firmly adhering to the maize roots) was removed using forceps, sun-dried, and sieved through a 2 mm sieve. Sieved rhizosphere soil samples were used for the extraction of bacterial genomic DNA for the evaluation of the bacterial diversity in soil and the determination of soil pH and U concentration.

### 2.2. Bacteria DNA Extraction and Gene Sequencing

Approximately 0.25 g of each rhizosphere soil sample was used for DNA extraction with the DNeasy^®^ PowerSoil^®^ Kit (Qiagen, Hilden, Germany), following the manufacturer’s protocol [34]. The extracted DNA was quantified using a Qubit™ 3.0 Fluorometer with the dsDNA High-Sensitivity assay and visualized via 1.0% agarose gel electrophoresis. The purified DNA was stored at −20 °C until further analysis [35]. For bacteriota analysis, the DNA samples were transported on dry ice to Inqaba Biotec™ in Pretoria, South Africa, where sequencing was conducted under run number 220803. The V3–V4 hypervariable regions of the 16S rRNA gene were amplified using the universal primer pair 341F (5′-CCTACGGGNGGCWGCAG-3′) and 785R (5′-GACTACHVGGGTATCTAATCC-3′). The resulting amplicons underwent gel purification, end-repair, and ligation with Illumina TruSeq adapters. Samples were then individually indexed, purified using bead-based methods, quantified, and pooled in equimolar concentrations. Finally, sequencing was performed on Illumina’s MiSeq platform using the MiSeq reagent kit v3 (600 cycles) [36].

### 2.3. Bioinformatics Analyses for the Soil Bacteriota Composition

Due to the low-quality scores observed in the reverse-end reads, bacteriota analyses primarily relied on forward reads. The decline in reverse-read quality was likely caused by the depletion of primers, dNTPs, and other reagents toward the end of the sequencing runs. Demultiplexed forward-end 16S rRNA gene reads were processed using the DADA2 pipeline (version 1.24.0) within R software (version 4.2.1) (R Core Team, 2022). The DADA2 workflow involved several steps, including read trimming and filtering, sequence dereplication, error rate learning, amplicon sequence variant (ASV) abundance table generation, and the removal of chimeric sequences using the “bimera de novo” method. The taxonomic classification of ASVs was conducted with the SILVA reference database (version 138). A total of 378,160 forward reads obtained from four bacterial DNA samples—one from each treatment—were processed in the DADA2 pipeline. Low-quality reads were removed by applying a truncation length of 240 bp and a left-trim threshold of less than 20 bp. The pipeline identified 6.8% of the total reads as chimeric, which were subsequently filtered out. This resulted in a final ASV abundance table containing 297,865 high-quality non-chimeric reads across the four samples. Therefore, the average sequencing depth per sample was 74,466 reads. Additionally, soil pH was measured using a calibrated pH meter (Orion VersaSTAR Pro, Thermo Fisher Scientific, Waltham, MA, USA) with a standard soil-to-water suspension ratio of 1:2.5.

### 2.4. Determination of U Concentration in Soil

The uranium (U) concentration in rhizosphere soil samples was measured using Energy-Dispersive X-ray Fluorescence (EDXRF) analysis, following a previously established method [37]. To ensure the accuracy and reliability of the results, the EDXRF spectrometer was validated using a commercially available standard reference material from the National Institute of Science and Technology (NIST 2711—Montana Soil, Moderately Elevated Trace Element Concentrations) and further verified with the International Atomic Energy Agency (IAEA) certified reference material (IAEA Soil 7—Trace Elements in Soil) (Table 1) [38,39]. The results showed an overall bias of ±5.8%, confirming that the measurement method met the required accuracy threshold (<10%) for this study.

### 2.5. Statistical Analysis

The statistical analysis of the relationship between soil pH and uranium (U) concentration was conducted using a one-way analysis of variance (ANOVA), with significant differences determined by Fisher’s LSD test at *p* < 0.05. All statistical tests were performed using STATISTICA version 8.0 (Stat-Soft, Inc., Tulsa, OK, USA). For sequence normalization, the ‘*phyloseq*’ package was used to analyze four treated soil samples. Sub-sampling was applied, retaining only highly abundant samples with a mean read count exceeding 1000. During this process, approximately 110 operational taxonomic units (OTUs) were removed, as they were absent from all samples following random sub-sampling. Alpha diversity indices, including Observed, Chao1, and Shannon, were employed to assess species richness and evenness at the phylum level across the four treatment plots, unfertilized (NF) and those fertilized with YC, NP, and MP. These indices were computed using the phyloseq package (version 1.40.0). Additionally, the distribution of unique, common, and shared amplicon sequence variants (ASVs) at the phylum, order, and class levels across the four treatments was visualized using the MicEco package (version 0.9.18) within R software (version 4.2.1) (R Core Team, 2022) [40].

## 3. Results

### 3.1. Effect of Soil pH and U Content in Different Fertilizer Application Plots

The results of the U concentrations detected in the fertilizers used in this study are shown in Table 2, and the accuracy of the measurements proved to show good precision (Table 2). The MP fertilizer had the highest U content (16,067 ± 48 mg kg^−1^), followed by the NP fertilizer (147.65 ± 8.61 mg kg^−1^), and the YC fertilizer had the lowest U content recorded at 38.84 ± 1.24 mg kg^−1^. Table 2 displays the results of pH and U concentrations in soil samples from the experimental plots, both before and after fertilizer application. Before maize planting, the soil pH and soil U content did not differ significantly among plots and averaged 5.22 ± 0.51 for pH and 2.02 ± 0.07 mg kg^−1^ for U. After maize planting and the application of fertilizers, several changes were observed among the plots. The soil pH in the control treatment (NF + M) and in the treatment YC + M did not change (Table 2). The pH of the soil rose significantly (*p* < 0.001) to 5.26 in the maize plot that was fertilized with Nafaka Plus (NP + M). In the maize plot that was fertilized with Minjingu Powder (MP + M), the pH rose slightly but not significantly (pH = 5.24). The amount of U in the soil rose sharply in the corn plot that was fertilized with NP (to 3.93 mg kg^−1^), then it rose slightly in the corn plot that was fertilized with MP (to 3.06 mg kg^−1^). The maize plot fertilized with YaraMila Cereal and the non-fertilized maize plot (control plot) did not differ significantly in soil pH and U content.

### 3.2. Effect of Soil N and P Content in Different Fertilizer Application Plots

Table 3 displays the soil N and P levels before and after fertilization. Before maize planting, soil N levels did not vary across the plots, ranging from 0.03 to 0.04%. However, soil P levels differed across the plots, ranging from 39.24 ± 0.00 to 40.18 ± 0.00 mg kg^−1^. We observed changes after maize planting and fertilization. The YC fertilizer had the highest nitrogen content at 0.07 ± 0.01%, the NP and MP fertilizer were next at 0.05 ± 0.01 and 0.04 ± 0.01%, respectively, while the NF (unfertilized) treatment had the lowest soil N levels at 0.02 ± 0.01%. On the other hand, after the experiment, soil P levels were significantly higher at 41.23 ± 0.01 mg kg^−1^ for the NP fertilizer, followed by the MP and YC fertilizers with 40.74 ± 0.01 and 40.59 ± 0.01 mg kg^−1^, respectively. The NF treatment had a significantly lower soil P level with 37.52 ± 0.01 mg kg^−1^.

### 3.3. Abundance of Soil Bacteria at Phylum Level in Maize Plots

**Figure 1:**
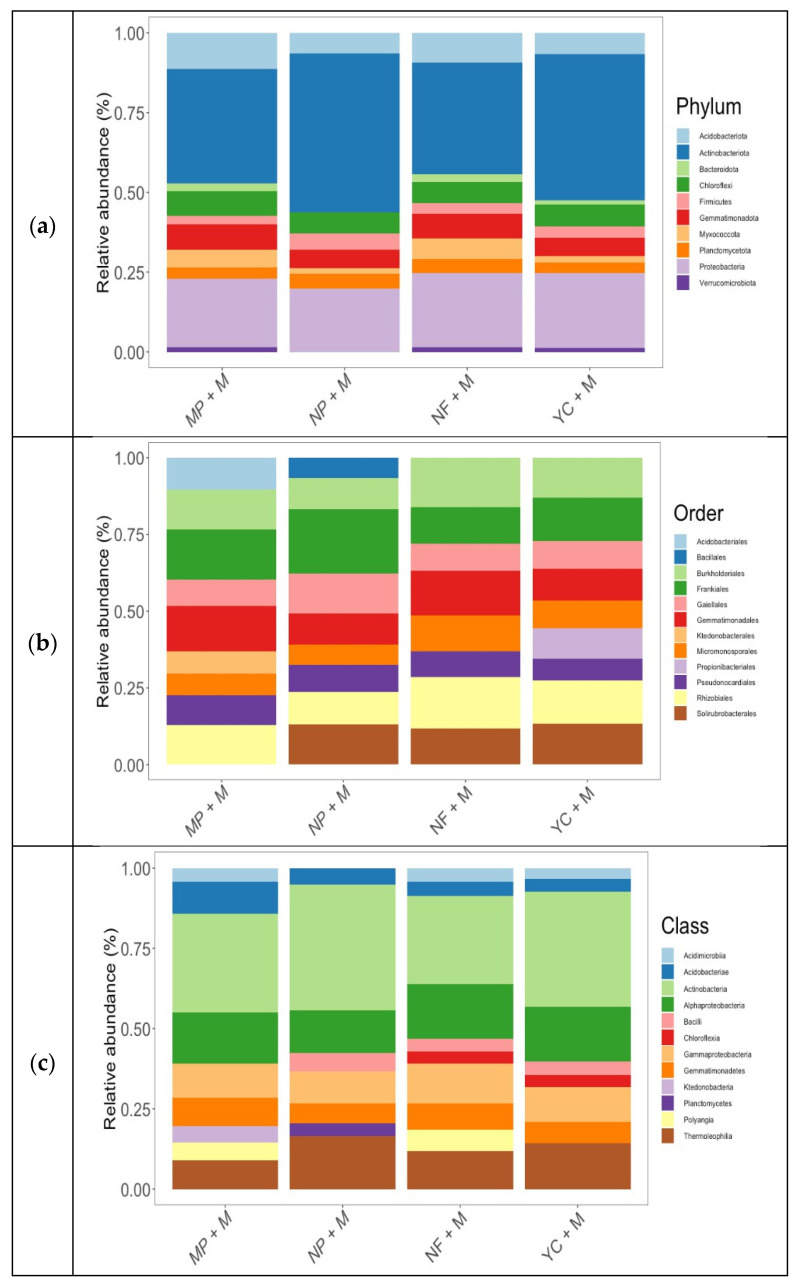
(**a**) Relative abundances (%) of soil bacterial phyla in each treatment applied to maize plantation, (**b**) relative abundances (%) of soil bacteria at order level for each treatment on maize plantation, and (**c**) relative abundance (%) of soil bacteria at class level for each treatment in maize plantation.

The total number of classifiable sequences was 297,865 and correlated with ten relative abundances of bacterial phyla from each treatment, i.e., MP + M, NP + M, NF + M, and YC + M (Figure 1a). The three main phyla in all treatments were *Actinobacteriota* (41.64%), *Proteobacteria* (22.0%), and *Acidobacteriota* (8.44%). Together, they made up 72.08% of all the phyla. Next to these phyla were *Chloroflexi* (7.00%), *Gemmatimonadota* (6.76%), *Planctomycetota* (4.02%), *Myxococcota* (3.96%), and *Firmicutes* (3.66%), followed by *Bacteroidota* (1.49%) and *Verrucomicrobiota* (1.01%). The relative abundance calculation did not include the bacterial phyla, which represented less than 1%.

The distribution of bacteria at the phylum level and their relative abundance varied with the treatments. There were a lot of *Actinobacteriota* bacteria in NP + M (12.42%), YC + M (11.50%), MP + M (8.98%), and NF + M (8.74%). *Proteobacteria* were abundant in YC + M (5.89%), followed by NF + M (5.82%), MP + M (5.35%), and NP + M (4.92%). Interestingly *Acidobacteriota* were found in MP + M (2.85%) and NF + M (2.32%), followed by YC + M (1.67%) and NP + M (1.59%). The plot that was fertilized with Minjingu Powder (MP + M) had 1.97% *Chloroflexi phylum*, 1.73% in YC + M, 1.66% in NF + M, and 1.65% in NP + M. The most *Gemmatimonodota* were found in the Minjingu Powder fertilizer (MP + M) plot (2.02%), then in the non-fertilized plot (NF + M) with 1.92%, then in the YC + M treatment (1.44%), and finally in the NP + M treatment (1.38%). There were 1.17% *Planctomycetota* in the NP + M fertilized plot, 1.08% in NF + M, 0.91% in MP + M, and 0.86% in YC + M. The non-fertilized plot (NF + M) had a higher abundance of *Myxococcota* (1.64%), followed by MP + M (1.36%), YC + M (0.98%), and NP + M (0.46%). *Firmicutes* were abundant in NP + M, reaching 1.29%, followed by YC + M at 0.91%, NF + M at 0.83%, and MP + M at 0.63%. *Bacteroidota* and *Verrucomicrobiota* phyla were very few; in an average of 1%, NF + M had 0.60 and 0.36% of *Bacteroidota* and *Verrucimicrobiota* phyla, respectively. YC + M had 0.31 and 0.29%, and MP + M had 0.58 and 0.36% of *Bacteroidota* and *Verrucimicrobiota* phyla, respectively.

### 3.4. Abundance of Soil Bacteria at Order Level in Maize Plots

*Frankiales* made up 15.91% of the soil bacteria in the maize treatments, followed by *Rhizobiales* with 13.47%, *Burkholderiales* with 12.96%, *Gemmatimonadales* with 12.20%, and *Gaiellales* with 10%. *Solirubrobacterales* (9.60%), *Micromonosporales* (8.56%), and *Pseudonocardiales* (8.49%) were the next most common orders after these five (Figure 1b). Furthermore, we detected the following orders in lower abundances: *Propionibacteriales* (2.63%), *Acidobacteriales* (2.55%), *Ktedonobacterales* (1.80%), and 1.74% of *Bacillales*.

Similarly to the relative abundance of bacteria at the phylum level, the abundance of bacteria at the order level also varied with the treatments. *Frankiales* were abundant in NP + M at 5.57%, followed by MP + M at 3.99%, YC + M at 3.75%, and NF + M at 2.60%. After *Frankiales*, *Rhizobiales* were abundant in YC + M (3.80%), followed by NF + M (3.69%), MP + M (3.15%), and NP + M (2.82%). *Burkholderiales* were rich in NF + M at 3.58%, YC + M at 3.50%, and MP + M at 3.18%. *Gemmatimonadales* were abundant in MP + M at 3.64%, followed by NF + M at 3.19%, YC + M at 2.73%, and NP + M at 2.65%. We found *Gaiellales* at a higher level in NP + M, reaching 3.45%, following by YC + M at 2.47%, MP + M at 2.12%, and NF + M at 1.98%. Other orders observed were *Solirubrobacterales* in YC + M (3.56%), NP + M (3.46%), and 2.63% of NF + M. *Micromonosporales*’ presence in decreasing abundance was 2.60% (NF + M), 2.46% (YC + M), 1.77% (NP + M), and 1.72% (MP + M), while *Pseudonocardiales* were abundant in MP + M at 2.40%, followed by NP + M at 2.32%, and YC + M at 1.90%. Only the YC + M treatment had 2.63% of the *Propionibacteriales* order, while only the MP + M treatment had 2.55% of the *Acidobacteriales* order and 1.80% of the *Ktedonobacterales* order. Finally, we found that only 1.74% of *Bacillales* were in the NP + M treatment.

### 3.5. Abundance of Soil Bacteria at Class Level in Maize Plots

Figure 1c displays the relative abundance of the twelve sequenced classes of soil bacteria. Another seven classes that were well represented were *Acidobacteriae* (5.68%), *Bacilli* (3.58%), *Gemmatimonadetes* (7.33%), *Thermoleophilia* (12.95%), *Gammaproteobacteria* (10.98%), *Acidobacteriae* (5.88%), and *Alphaproteobacteria* (15.81%). Other classes in lesser abundance were *Polyangia* (3.02%), *Acidimicrobiia* (2.92%), *Chloroflexia* (1.81%), *Ktedonobacteria* (1.22%), and *Planctomycetes* (1.02%). Six classes were present in all treatments: *Actinobacteria*, *Alphaproteobacteria*, *Thermoleophilia*, *Gammaproteobacteria*, *Gemmatimonadetes,* and *Acidobacteriae*. The treatment with Nafaka Plus (NP + M) displayed the highest abundance of the *Actinobacteria* class (10.11%), followed by the YC + M treatment with 9.11%, the MP + M treatment with 7.55%, and the NF + M treatment with 6.59%. It was NF + M that had the most *Alphaproteobacteria* (4.08%), then MP + M at 3.92%, and finally NP + M at 3.43%. The abundance of the *Thermoleophilia* class was higher in the NP + M treatment, reaching 4.24%, followed by YC + M at 3.67%, NF + M at 2.82%, and MP + M at 2.21%. The *Gammaproteobacteria* class was abundant in NF + M at 3.01%, YC + M at 2.78%, MP + M at 2.63%, and NP + M at 2.56%. The MP + M had 2.22% of the *Gemmatimonadetes* class, followed by NF + M at 1.94%, YC + M at 1.66%, and NP + M at 1.62%. The *Acidobacteriae* class had 2.44% in the MP + M treatment, and next to it was the NP + M treatment with 1.32%, followed by NF + M at 1.10% and YC + M at 1.02%. The abundance of *Bacilli* was observed in NP + M at 1.51%, followed by YC + M at 1.08% and NF + M at 0.99%. Only NF + M and MP + M exhibited the *Polyangia* class, with 1.65% and 1.37%, respectively. In MP + M, NF + M, and YC + M, the *Acidimicrobiia* class was observed at 1.05, 0.99, and 0.87%, respectively. *Chloroflexia* was abundant at 0.94 and 0.86% in the YC + M and NF + M treatments, respectively. The *Ktedonobacteria* class was only found in the MP + M treatment at 1.22%, while the *Planctomycetes* class was abundant in the NP + M treatment at 1.03%.

### 3.6. Composition of Phylum Community Variation Within Treatments

**Figure 2:**
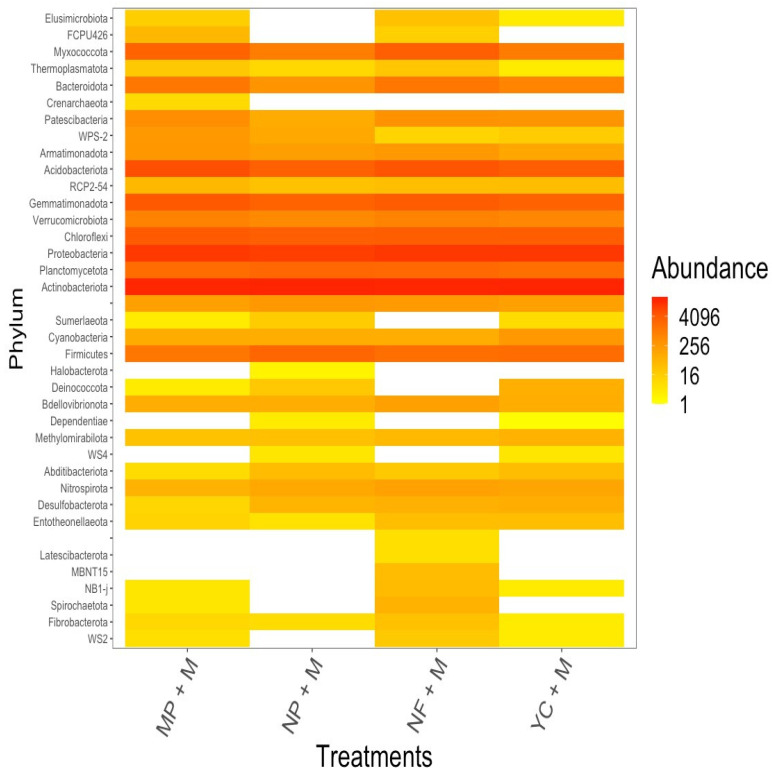
Heat map indicating the absolute abundance of bacteria phyla in maize treatments: fertilized with Minjingu Powder (MP + M), fertilized with Nafaka Plus (NP + M), not fertilized (NF + M), and fertilized with YaraMila Cereal (YC + M).

In Figure 2, the total phyla seen in the maize treatments are shown as a heat map. This includes the phyla with less than 1%. The most abundant phyla are indicated in red; the least represented phyla are indicated in yellow, and the white color denotes no phyla observed (Figure 2). Among the least abundant phyla (yellow), Abditibacteriota, Entotheonellaeota, Fibrobacterota, and Thermoplasmatota were sparsely detected across treatments. There were *Spirochaetota* in the MP + M, NF + M, and YC + M treatments, but only *Elusimicrobiota* in the MP + M, NF + M, and YC + M treatments. Only the MP + M treatment showed *Crenarchaeota*, while only the NP + M treatment showed *Halobacteriota*. Only the NF + M treatment showed Latescibacterota, MBNT-15, and NB1-j. The most common phyla were the same as in Figure 1: *Actinobacteriota*, *Proteobacteria*, *Acidobacteriota*, *Chloroflexi*, *Gemmatimonadota*, *Planctomycetota*, *Myxococcota*, *Firmicutes*, *Bacteroidota*, *Verrucomicrobiota*, *Armatimonodota*, and *Patescibacteria*.

### 3.7. Venn Diagram Analysis for Unique Phyla, Classes, and Orders of Bacteria

**Figure 3:**
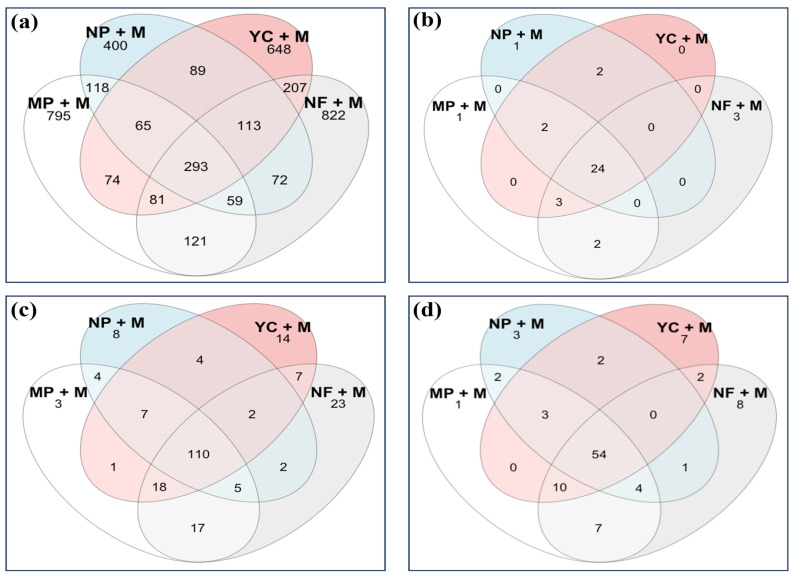
Venn diagram showing operational taxonomic units (**a**) OTUs, (**b**) phylum levels, (**c**) bacterial orders, and (**d**) bacterial classes of unfertilized (NF + M), YaraMila Cereal (YC + M), Nafaka Plus (NP + M), and Minjingu Powder (MP + M) treatments.

The Venn diagram detected the number of common and unique bacterial species in all treatments (Figure 3a). The results indicated that 293 species of operational taxonomic units (OTUs) (7.40% of all OTUs) were common to all treatments. The NF + M treatment had the highest number of bacterial OTUs, reaching 822, and accounting for 20.77% of the total. The MP + M was second, with unique OTUs reaching 795, equivalent to 20.09% of the total OTU samples. The YaraMila Cereal treatment (YC + M) had 648 unique OTUs (16.38%), while NP + M had 400 OTUs, accounting for 10.11%.

Soil bacteria from all treatments belonged to a total of 31 phyla. A Venn diagram (Figure 3b) illustrates the number of shared phyla across all treatments. All maize treatments shared 24 phyla, while 3 were unique to the NF + M treatment, accounting for 2.42% of the total phyla. In addition, the Venn diagram shows one phylum unique to the MP + M and another one phylum unique to NP + M, accounting for 0.81% in each case. For the YC + M treatment, no unique phylum was observed. A total of 656 bacterial orders were identified in all the maize treatments (Figure 3c). All treatments shared 110 bacterial orders, which accounted for 16.77% of the total. The most abundant orders recorded were in the NF + M treatment with 23 orders, equivalent to 3.51% of the total, followed by the YC + M treatment, with 14 unique orders, accounting for 2.13%. The NP + M treatment had eight unique orders, equivalent to 1.22% of the total. The lowest number of bacterial orders was recorded in the MP + M treatment, with only three orders, accounting for 0.46%. The abundance of soil bacteria at the class level in all the treatments is visualized in a Venn diagram (Figure 3d). The number of bacterial classes at each intersection of the four treatments was 54, accounting for 17.20% of the total. The NF + M treatment had the highest number of bacterial classes (eight), equivalent to 2.55%, followed by YC + M, equivalent to 2.23%. The NP + M had three bacterial classes, equivalent to 0.95%, and the MP + M treatment had only one unique class of bacteria that accounted for 0.32%.

### 3.8. Observed, Chao1, and Shannon Diversity Indices Indicating Phylum-Level Diversity Across Treatments

**Figure 4:**
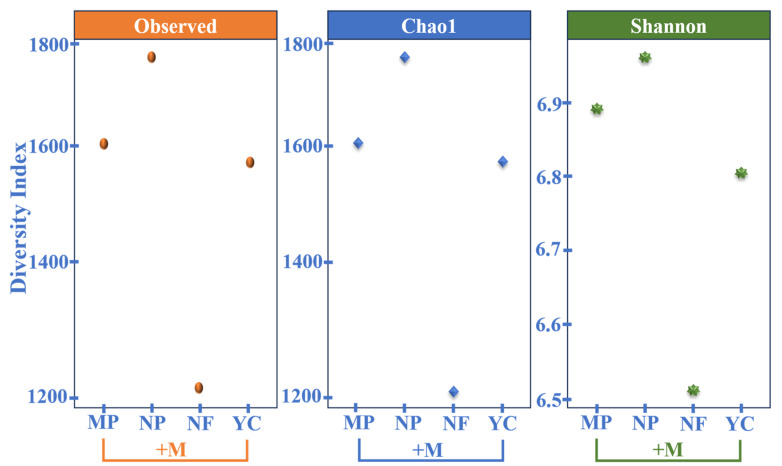
Diversity indices of species richness (Observed and Chao1), and evenness (Shannon) at phylum level in treatments: non-fertilized (NF + M), YaraMila Cereal (YC + M), Nafaka Plus (NP + M), and Minjingu Powder (MP + M) treatments.

The Observed and Chao1 indices show no significant change in bacteria phyla and their proportions. However, the Shannon diversity index indicates a slight change in bacterial diversity (Figure 4).

The Shannon index indicates a diversity change while the other indices (Observed and Chao1) show similar patterns. This can be explained due to each index’s sensitivity and underlying mathematical properties. The Observed index measures species richness by counting different unique taxa present in the samples without considering their abundances. If species’ richness, rather than their relative abundances, contributes to the variations, it does not demonstrate significant changes. The Chao1 index measures species richness similarly to the Observed index, except that it only counts unique, rare taxa and assumes that many taxa are present but not observed. It is less sensitive to changes in the relative abundances of taxa. The Shannon index considers species richness and evenness by assigning higher weights to abundant and rare taxa. It is very sensitive to diversity changes associated with the relative abundances of taxa.

## 4. Discussion

### 4.1. Rhizosphere Change in U Content and Soil pH Following Application of Different Types of Fertilizer

The non-fertilized plot (NF + M) had the highest total operational taxonomic units (OTUs), reaching 822, followed by the MP + M and YC + M plots, which had 795 and 648 amplicon sequence variants (ASVs), respectively. The higher the U concentration in the soil, the lower the number of ASVs. The NP fertilizer application in maize plots produced a significant (*p* < 0.001) increase in U content in soil to 3.93 mg kg^−1^, indicating that the fertilizer had a substantial content of U derived from the MPR. On the other hand, maize plots fertilized with MP (the natural rock in powder form used as fertilizer) had the lowest soil U concentration of 3.06 mg kg^−1^, which was lower by 0.87 units compared to the NP fertilizer (Table 2). This indicates that MP, despite its content having a higher U concentration, had a lower U concentration in soil compared to NP because most of its U concentration could have been easily lost through leaching as it was applied in powder form. The organic carbon and calcium content of the soil under the study site was very low, which could have resulted in the low retention of U released by NP and MP in soils. Following fertilizer applications, soil pH was increased in two treatments, T3 and T4. This pH increase was attributed to the CaO content of fertilizers, which was 36% and 25% for the MP and NP fertilizers, respectively (Table 1). The U concentrations in rhizosphere soils also increased in the treatments with these fertilizers and was associated with the soil pH increase in the NP + M and MP + M treatments (Table 2). Soil pH is the most determinant factor for the U distribution coefficient as it increases to pH 6 [16,41,42,43]. Research by Mondani et al. [18], Islam & Sar [44], Yan & Luo [45], and Li et al. [13] further indicates that uranium and soil pH consistently put a lot of stress on soil bacterial communities. The non-existent change in soil pH and U content observed in non-fertilized (NF) plots and YC-fertilized plots further signifies that the YC fertilizer has minor U impurities.

### 4.2. Distribution of Soil Bacteria in Plots Fertilized with Different Kinds of Fertilizer

The higher U residual contents in soils due to fertilization with NP and MP showed an abundance of bacterial diversity. The higher U was observed to enhance the abundance of bacterial diversity in the NP + M treatment (Figure 4). The *Actinobacteriota* phylum was mainly abundant across the treatments (Figure 1a). However, the NP + M treatment showed more abundance, followed closely by the YC + M treatment, indicating that a greater amount of nutrient elements in these fertilizers could have favored the *Actinobacteriota* phylum by 12.42% (Figure 1a). NP + M, besides having N, P, K, S, Mg, and Zn, which were also found in YC + M, also had Ca and B nutrients [33]. After NP + M, the MP + M treatment was found to release the most content of U in soils, composed mainly of P, Ca, and, to a lesser extent, Mg [33], and had 8.98% of the *Actinobacteriota* phylum, which was a bit higher than that of NF + M, at 8.74%. The *Proteobacteria* phylum showed a uniform distribution trend across the treatments, proving to be very stable in different types of fertilizer applications.

The NF + M treatment had a higher number of unique bacterial orders and classes, followed by YC + M, NP + M, and MP + M. The lower number of bacterial orders and classes in NP + M and MP + M could be related to their U residual contents, which negatively affected the bacterial orders compared to the YC + M treatment (Figure 3c,d). The top three orders of soil bacteria across the treatments were *Frankiales* (15.91%), *Rhizobiales* (13.47%), and *Burkholderiales* (12.96%). The abundance of the *Frankiales* bacterial order, related to its role of fixing nitrogen to the maize rhizosphere, was highest in NP + M and MP + M, followed by the YC + M and NF + M treatments (Figure 1b). The *Pseudonocardiales* order in the class *Actinobacteria* was most abundant in the MP + M and NP + M plots, implying that these fertilizers with U impurities favored mostly the class of *Actinobacteria* (Figure 1b,c).

Various studies have observed that *Actinobacteria* possess different genes with abilities to help them withstand abiotic stress, produce secondary metabolites, and resist metals, which allows them to change uranium into uranyl phosphate minerals and play a role in the cycling of uranium in the environment [46]. Furthermore, *Actinobacteria* have been observed to actively produce essential natural components including organic compounds, generate various organic acids, and lower pH to solubilize phosphate salts and produce phosphatase, thereby making phosphorus available to plants [47,48,49,50,51]. On the other hand, NP + M was also rich in *N.* The *Bacillales* order in the class of *Bacilli* was rich in the NP + M treatment only (Figure 1b), which was also found to have high U soil residual (Table 2). *Bacilli*, like *Pseudonocardiales*, are prevalent in soil with elevated uranium levels, potentially associated with universal stress proteins (USPs). These proteins help bacteria adapt to their soil surroundings and are important for forming biofilms [52]. The USPs play crucial roles in cellular responses to biotic and abiotic stresses, whereby they have been observed to be involved in cell growth and development [53]. The *Acidobacteriales* order in the class of *Acidobacteriia* and the *Ktedonobacterales* order in the class of *Ktedonobacteria* were only rich in the MP + M treatment, indicating that they could have a role in soil P cycling and regulation of P availability in soil [54,55,56]. The *Chloroflexia* class was not found in MP + M and NP + M (Figure 1c) as these fertilizer treatments have a substantial amount of P.

The bacterial diversity in the treatments was observed to be higher than that in the unfertilized treatments (Figure 4), indicating that the bacterial species abundance and richness were influenced by fertilization (Figure 3b–d) and increased their activity in soils. The current research showed that soils fertilized with higher U residuals had a lot of *Actinobacteriota*, *Proteobacteria*, *Acidobacteriota*, *Chloroflexi*, *Gemmatimonadota*, *Planctomycetota*, *Myxococcota*, *Firmicutes*, and *Bacteroidota*. More types and amounts of bacteria were found in NP + M, which made it easier for bacteria to grow because more U was released into the soil. The *Actinobacteriota* phylum was unique and abundant in NP + M. Contrarily, the MP + M treatment had somewhat lower bacteria richness and diversity, as its released U in soil was lower than that of the NP + M treatment, and *Acidobacteriota* were unique and abundant in MP + M (Figure 1a and Figure 3b). Also, the YC + M fertilizer treatment had the least amount of U that did not affect anything else in the soil, so it had the fewest types and amounts of bacteria. This is likely because it did not have any effect that would have changed the bacteria’s environment. *Crenarchaeota* were found only in the MP + M treatment with its higher content of U (Figure 3a and Figure 4). In addition, small changes were observed in soil pH after fertilization (Table 2), suggesting that this group is sensitive to changes in soil pH, possibly due to the amount of calcium present. *Halobacteriota* were found only in the NP + M treatment (Figure 3a and Figure 4), indicating that it tolerated a higher U concentration, but it also could be involved in the cycling of S content in soils as the NP fertilizer has a higher percentage of sulfur content [33]. In addition, *Halobacteriota* are known to thrive in environments with high salt and high levels of magnesium and calcium [57,58]; therefore, using fertilizers that contain CaSO_4_, like NP, might explain why they were found in the NP + M treatment. The occurrence of *Halobacteriota* and *Crenarchaeota* in NP and MP, respectively, indicates that these fertilizer products may facilitate uranium retention through calcium-containing compounds (Table 2 and [59]). The unfertilized treatment (NF + M) had a significantly lower bacteria richness and diversity, indicating that not adding nutrients to soils does not affect bacterial richness and diversity. *Latescibacterota*, *MBNT-15,* and *NB1-j* were unique (Figure 3a and Figure 4) and only found in the unfertilized plot (NF + M), showing that the lack of fertilization promotes natural rhizosphere microbial interactions. Despite all these findings, further research studies are needed to explore the agronomic role of *Latescibacterota* and other unidentified bacteria under the unfertilized regime.

Despite pH and U impacting diversity, significant changes in soil P and N after fertilization (Table 3) may also be linked to shifts in bacterial diversity. Other studies by Delgado-Baquerizo et al. [60] revealed that soil N and P nutrients are strong predictors of bacterial diversity and composition. Soil N and P have the greatest observed influences on soil bacterial and fungal diversity [61]. The addition of N through fertilization affects the bacterial community primarily by changing the pH; thus, fertilizer added in soil with N nutrients could have contributed to the bacterial communities, as similarly observed by Craig et al. [62] and Beltran-Garcia et al. [63]. On the other hand, the addition of P through fertilization affects the bacterial community primarily via mediating P availability [64]. A study by Wang et al. [65] revealed that P in soil significantly increased the relative abundance of plant-beneficial bacteria, particularly *Firmicutes*, *Nitrospirae*, and *Acidobacteria*. Further, they observed that the abundance of *Bacillus*, belonging to *Firmicutes*, was dramatically increased.

### 4.3. Limitations

This pilot study on uranium in agricultural soils has several limitations. It was conducted at a single field site with low organic carbon and calcium levels, which may not be representative of other agricultural soils. Fertilizer treatments varied in U content and nutrient composition, potentially introducing confounding factors. This study also spanned a single cropping season with moderate U accumulation, which may not capture long-term effects of uranium on the soil microbiome. Longer-term field trials or chronosequence studies would help determine if initial diversity increases observed at moderate U levels persist, are reversed, or lead to functional impairments over time [66].

The microbiological analysis had limitations, with limited sample replication and limited functional information. The study also did not measure important soil health indicators, such as microbial biomass or enzymatic activities. Future work could incorporate assays of microbial activity and soil health to better understand how fertilizer-derived U influences soil ecosystem function [66].

Our study assumed the soil moisture to be constant in all treatments, ensuring that it did not influence the relative results. However, we recognize that this could be a limitation and suggest that further studies should include soil moisture monitoring to better understand its moderating role. Environmental variability and long-term effects are also significant factors that should be considered in future studies across different soil types (environment) and field conditions to validate and expand upon our results.

## 5. Conclusions

The phosphate fertilizer derived from MP shows particularly high U levels, and the application of this fertilizer increased the concentration of U in the investigated soil to 3.06–3.93 mg kg^−1^ and the soil pH to 5.26. The increase in U in the examined soils was observed to increase bacterial abundance and diversity. However, *Halobacteriota*, in their lowest abundance, were unique to NP + M due to their ability to tolerate higher U concentrations and high CaSO_4_. *Crenarchaeota*, in their lowest abundance, were unique in MP + M, with its lesser U concentration in comparison to the NP + M treatment, due to their ability to persist in heavy metals. Under the unfertilized treatment (NF + M), *Latescibacterota*, MBNT-15, and NB1-j demonstrated exceptional uniqueness, suggesting their exclusive availability to unfertilized plots. The treatment with the highest amount of U in the soil, 3.93 mg kg^−1^, had an abundance of the *Bacillales* order, which is in the *Bacilli* class. It stayed the same, which indicates that it could handle the higher U concentration. Also, a lot of the *Pseudonocardiales* order, which is part of the *Actinobacteria* class, could be seen in the MP + M and NP + M plots. This suggests that they can handle higher U concentrations. The *Rhizobiales* order in the *Alphaproteobacteria* class, on the other hand, was abundant in YC + M where not much U was released in the soil. The current study suggests that phosphate fertilizer releases U in soils and its contents could accumulate in soils following longer-term application. Therefore, it is more likely that using these fertilizers for a longer time will raise the U levels in the soil, change the types of bacteria that live there, and cause more activity in the rhizosphere. However, the bacterial diversity may also be responsible for the changes in soil P and N after fertilization.

Therefore, future studies should consider the effects of soil moisture dynamics, environmental variability, and long-term temporal factors, replicated across diverse soil types and field conditions, to robustly validate the observed patterns in bacterial diversity.

## Figures and Tables

**Table 1:** Composition of fertilizers used in the experiments along with measured U mass concentration in IAEA-Soil 7 and other certified reference material.

Treatment Code	Fertilizer/Treatment Component	Fertilizer Formulation	Uranium Concentration (mg kg^−1^)
T1	Not fertilized (NF + M)	No fertilizer used	-
T2	YaraMila Cereal (YC + M)	23N:10P:5K + 3S + 2MgO + 0.3Zn	38.84 ± 1.24
T3	Nafaka Plus (NP + M)	N: 9%, P_2_O_5_: 16%, K_2_O: 6%, CaO: 25%, S: 5%, MgO: 2%, Zn: 0.5%, B: 0.1%	147.65 ± 8.61
T4	Minjingu Powder (MP + M)	P_2_O_5_: 28%	159.67 ± 10.48
**Measured Uranium Mass Concentration**			
**Reference Material**	**Uranium Concentration (mg kg^−1^)**		**Bias (%)**
	**Measured**	**Reference**	
**IAEA Soil 7**	2.78 ± 0.43	2.6 ± 0.6	5.8%
**NIST 2711a**	3.10	2.96	5.1%

M = maize plantation.

**Table 2:** Soil pH and uranium content before and after fertilization.

Treatments (T)	Soil pH Before Fertilization	Soil pH After Fertilization	Soil U (mg kg^−1^) Before Fertilization	Soil U (mg kg^−1^) After Fertilization
T1 = NF + M	5.21 ± 0.01 ^a^	5.21 ± 0.01 ^c^	2.04 ± 0.01 ^a^	2.04 ± 0.02 ^c^
T2 = YC + M	5.22 ± 0.00 ^a^	5.22 ± 0.01 ^bc^	2.02 ± 0.02 ^a^	2.04 ± 0.01 ^c^
T3 = NP + M	5.22 ± 0.00 ^a^	5.26 ± 0.01 ^a^	2.01 ± 0.02 ^a^	3.93 ± 0.28 ^a^
T4 = MP + M	5.22 ± 0.00 ^a^	5.24 ± 0.01 ^ab^	2.02 ± 0.01 ^a^	3.06 ± 0.15 ^b^
F-statistics	0.27 ^ns^	0.02 *	0.48 ^ns^	0.00 ***

Means in the same category of evaluated interface sharing similar letter(s) do not differ significantly (ns = not significant) based on their respective standard error (SE) at a 5% error rate. Values presented are means ± SE $\bar{x}$ (standard error of means); * and *** mean significant at *p* < 0.05 and *p* < 0.001.

**Table 3:** Soil nutrient levels for N and P before and after the trials.

Treatments (Ts)	Soil N Before Fertilization (%)	Soil N After Fertilization (%)	Soil P Before Fertilization (mg kg^−1^)	Soil P After Fertilization (mg kg^−1^)
T1 = NF + M	0.03 ± 0.00 ^a^	0.02 ± 0.01 ^c^	39.24 ± 0.00 ^d^	37.52 ± 0.01 ^d^
T2 = YC + M	0.04 ± 0.00 ^a^	0.07 ± 0.01 ^a^	39.97 ± 0.00 ^b^	40.59 ± 0.01 ^c^
T3 = NP + M	0.04 ± 0.00 ^a^	0.05 ± 0.01 ^b^	40.18 ± 0.00 ^a^	41.23 ± 0.01 ^a^
T4 = MP + M	0.04 ± 0.00 ^a^	0.04 ± 0.01 ^b^	39.91 ± 0.00 ^c^	40.74 ± 0.01 ^b^
F-statistics	1.53 ^ns^	20.31 ***	6864 ***	105,647 ***

Means in the same category of evaluated interface sharing similar letter(s) do not differ significantly (ns = not significant) based on their respective standard error (SE) at 5% error rate. Values presented are means ± SE $\bar{x}$ (standard error of means); *** means significant at *p* < 0.001.

## Data Availability

The original contributions presented in this study are included in the article. Further inquiries can be directed to the corresponding author.
